# Variety Wins: Soccer-Playing Robots and Infant Walking

**DOI:** 10.3389/fnbot.2018.00019

**Published:** 2018-05-09

**Authors:** Ori Ossmy, Justine E. Hoch, Patrick MacAlpine, Shohan Hasan, Peter Stone, Karen E. Adolph

**Affiliations:** ^1^Department of Psychology, New York University, New York, NY, United States; ^2^Department of Computer Science, University of Texas at Austin, Austin, TX, United States

**Keywords:** infant walking, locomotion, bipedal robotics, robot soccer, natural gait

## Abstract

Although both infancy and artificial intelligence (AI) researchers are interested in developing systems that produce adaptive, functional behavior, the two disciplines rarely capitalize on their complementary expertise. Here, we used soccer-playing robots to test a central question about the development of infant walking. During natural activity, infants' locomotor paths are immensely varied. They walk along curved, multi-directional paths with frequent starts and stops. Is the variability observed in spontaneous infant walking a “feature” or a “bug?” In other words, is variability beneficial for functional walking performance? To address this question, we trained soccer-playing robots on walking paths generated by infants during free play and tested them in simulated games of “RoboCup.” In Tournament 1, we compared the functional performance of a simulated robot soccer team trained on infants' natural paths with teams trained on less varied, geometric paths—straight lines, circles, and squares. Across 1,000 head-to-head simulated soccer matches, the infant-trained team consistently beat all teams trained with less varied walking paths. In Tournament 2, we compared teams trained on different clusters of infant walking paths. The team trained with the most varied combination of path shape, step direction, number of steps, and number of starts and stops outperformed teams trained with less varied paths. This evidence indicates that variety is a crucial feature supporting functional walking performance. More generally, we propose that robotics provides a fruitful avenue for testing hypotheses about infant development; reciprocally, observations of infant behavior may inform research on artificial intelligence.

## Introduction

Both infancy and artificial intelligence (AI) researchers are interested in developing systems that produce adaptive, functional behavior. Infancy researchers have the benefit of starting with infants—one of nature's most flexible and generative learning machines. Through observation, infancy researchers work backward to reverse engineer infants' underlying learning mechanisms and develop formal theories. These theories, however, are often difficult to test experimentally; controlled rearing environments and training regimens are notoriously slow, burdensome, and in some cases, outright impossible. AI researchers have the benefit of building models, but can gain insights into the processes of change by studying natural learning systems (Gómez et al., 2004; Cangelosi et al., 2015). Here, we use the computational power of AI to test an otherwise intractable developmental question: What is the best way to learn a generative skill like walking?

## Variety in spontaneous infant walking: a feature or a bug?

Variety is essential for functional motor behavior. Movements must be tailored to the changing constraints of the body, environment, and task (Gibson, 1979; Newell, 1986; Bernstein, 1996). Functional walking, for example, is a highly creative process. It requires more than alternating leg movements to get from A to B. No step is ever repeated in exactly the same way or under exactly the same conditions. To successfully navigate the environment, walking must be continually modified to suit changes in local conditions—different surfaces (e.g., walking on pavement or sand), changes in layout (e.g., walking uphill or over flat ground), and obstacles along the path (e.g., clutter, elevations, and other agents who move). Thus, functional walking requires agents to navigate varied paths to adapt to moment-to-moment changes in body-environment relations (Adolph, 2008; Adolph and Robinson, 2015). How does anyone, let alone an infant, learn such a generative skill? What sort of training regimen facilitates the acquisition of flexible, creative, adaptive motor action?

Decades of research on the development of walking have focused on the acquisition of periodic gait—the ability to maintain steady-state velocity in a straight line using a series of alternating steps (Adolph et al., 2003; Ivanenko et al., 2004; Chang et al., 2006; Hallemans et al., 2006; Bisi and Stagni, 2015; Bril et al., 2015). With straight-line walking as the “gold standard,” research on motor learning and rehabilitation has focused on training uniform, alternating steps (Cherng et al., 2007; Ivanenko et al., 2007; Ulrich et al., 2008; Reisman et al., 2009; Willoughby et al., 2010). Although such training leads to improvements in strength, and indeed improvements in straight-line walking, it does little to improve the functional, flexible, adaptive, walking skills needed to navigate a real-world environment. So, what does? A growing literature on motor learning recognizes the beneficial role of variable practice (Moxley, 1979; Catalano and Kleiner, 1984; Van Rossum, 1990; Schmidt, 2003; Davids et al., 2006; Ranganathan and Newell, 2013). The principle at the heart of this line of research is that more variability in practice leads to greater flexibility outside the training environment.

Initially, infant walking is highly variable. Infants' gait is inconsistent from step to step (Clark et al., 1988; Bonneuil and Bril, 2012). Infants cannot reproduce leg movements consistently, they cannot walk quickly, they cannot walk far, and they fall a lot (Adolph et al., 2012). New walkers are bad walkers, but they get better with experience (Adolph and Robinson, 2015). Moreover, individual infants display a tremendous variety of path shapes during spontaneous walking in free play. They produce both short and long bouts; they generate curving, serpentine, and zigzag paths; they double back on themselves; they step in every direction and sometimes take multiple steps on the same foot (Adolph et al., 2012; Lee et al., 2017). These varied paths steer infants around toys and people, but infants also take varied paths over open ground, when nothing is in the way (Hoch et al., 2017).

Is the variety in infant walking paths a feature or a bug? If variety is a feature, then infants' early experience with varied walking paths may be beneficial for learning functional walking. If variety is a bug, then infants' varied paths may add noise that impedes or, at best, has no consequences for learning. More likely, it is both. Learning on varied walking paths presumably has both costs and benefits depending on the task. Recent work suggests that early experience with varied walking paths may be an essential component of infants' natural training regimen. Short bouts, curving paths, and omnidirectional steps are endemic from infants' first steps until many months after walk onset (Lee et al., 2017). Inconsistency goes away with walking experience. Varied paths do not.

## Humanoid robots learning to walk: RoboCup!

Much like infants, for robots, functional movement in a realistic physical environment (simulated or real world) requires a behavioral flexibility. In the robot world, successful, functional locomotor performance is assessed with robot soccer competitions. Why soccer? Historically, computer scientists believed that a truly intelligent artificial agent might be able to beat a human at chess (1997; Deep Blue), at trivia (2011; Watson), or more complicated strategy games (2017; Alpha-Go). However, in 1997, the same year Deep Blue defeated chess grandmaster and former world chess champion Garry Kasparov, a new breed of AI researchers decided that rather than learning and implementing a set of rules, true intelligence might look something more like generative, adaptive, embodied motor action. To meet this challenge, they created RoboCup—the world's premier robot soccer competition (Visser and Burkhard, 2007). The original call of the RoboCup initiative was to create a team of autonomous humanoid robots that could beat the human soccer world cup champions by the year 2050 (Kitano et al., 1997; Burkhard et al., 2002).

Soccer competitions are a good measure of functional locomotor performance because players cannot simply enact a set of rules or merely produce repetitive movements. Seeing many “moves” into the future, as in chess, is not sufficient. Instead, soccer players must take rapid steps in every direction along curved and sharply turning paths—all while the locations of the ball, players on both teams, and the relative positions of the goals are changing. Thus, soccer-playing robots, like infants, must learn in a way that facilitates flexible, goal-directed locomotion in a continually changing environment.

Previous studies showed that training robots with omnidirectional walking paths decreased falls and increased speed and distance traveled, leading to smoother and faster turns compared to training on unidirectional walking (Urieli et al., 2011). Likewise, training robots on infants' walking paths may improve robots' locomotor performance.

## Current studies

In the current studies, we used simulated soccer-playing robots as a model system to ask whether infants' naturally varied walking paths are beneficial for learning functional walking. Although the full variety of infants' walking experiences is unknown, the quantity is massive. Infants take an estimated 2,400 steps and travel the length of 7.7 American football fields in 1 h of free play with caregivers (Adolph et al., 2012). Thus, any experimental training regimen with infants would likely be swamped by the sheer quantity of their everyday experiences. Given that it is not feasible to control infants' everyday walking experience (or even record their walking paths over a waking day), we exploited the computational power of RoboCup to experimentally test the hypothesis that paths varying in shape, step direction, number of steps, and number of starts and stops are better training for functional walking than less varied paths. Specifically, we compared the outcomes of different robot training regimens using simulated robot-soccer competitions. By using simulated robots as models of real-world infant walking, we could control the training regimen and obtain robust estimates of performance over thousands of games of RoboCup. In the current studies, we aimed to: (1) experimentally examine the role of varied paths in learning functional walking, and (2) test whether differences in the natural variety of infant walking paths affect functional performance. We addressed these aims in two simulated robot soccer tournaments.

To address our first aim, in Tournament 1, we trained one team of robots on a training course composed of infants' natural—and highly varied—walking paths. The “opposing” teams were trained using uniform geometric paths: straight-lines, squares, and circles. To evaluate the success of the different training regimens, each pair of teams played off in a series of head-to-head soccer games. We predicted that the robot team trained on infant paths would outperform the teams trained on less variable geometric paths (infant-trained robots would score more goals and win more games).

To address our second aim, in Tournament 2, we compared robots trained on infant walking paths that varied in several aspects—shape, step direction, number of steps, and number of starts and stops. Variety in path shape—some straighter and some curvier paths—reflects the ability to control the two sides of the body independently. Variety in step direction—forward, backward, and sideways—reflects the ability to produce steps in every direction. Variety in the number of steps reflects the ability to produce both short and long bouts of locomotion. Finally, the number of starts and stops reflects the ability to initiate and control disequilibrium. We clustered infants into five groups based on these measures of path variety and trained soccer teams according to the five sets of paths. It is important to note that soccer involves more than just walking. Players must also have the ability to kick the ball and collaborate with others. However, because the current studies focus on walking, all other skills remained constant and equal across teams. Therefore, if one team performed significantly better than another, the advantage was due to differences in walking training.

## General methods

### Infant walking paths

**Figure 1:**
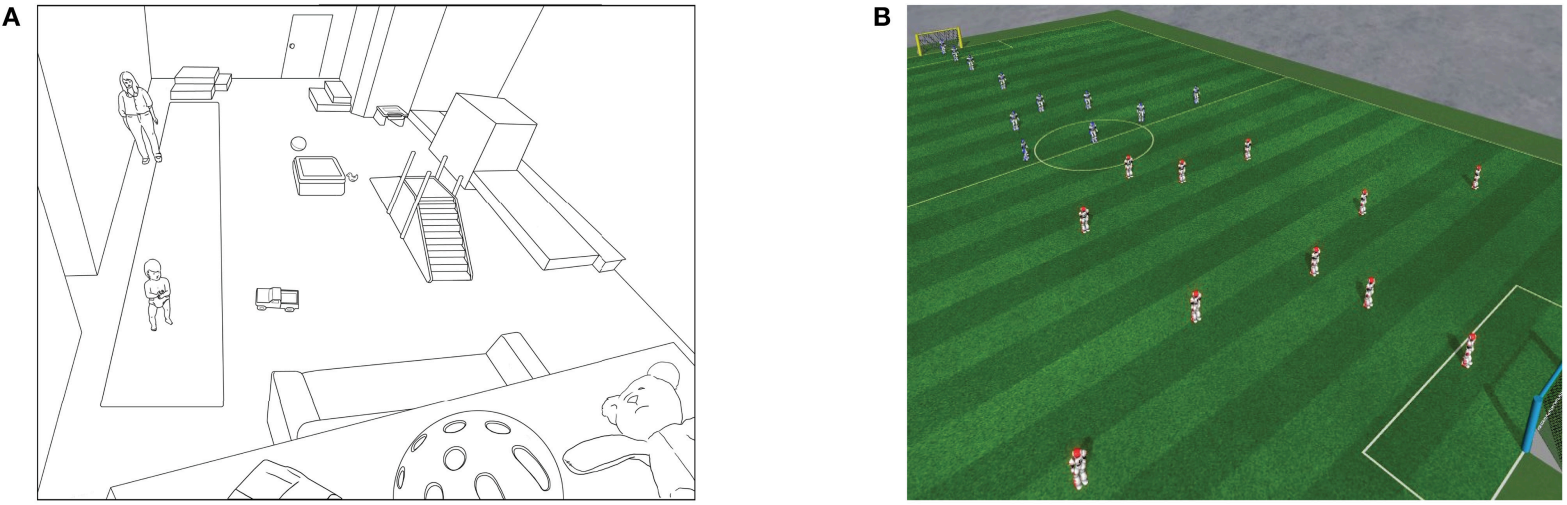
**(A)** Layout of the laboratory playroom. **(B)** Simulated RoboCup soccer field.

We observed the walking paths of 90 infants (49 girls, 41 boys) from the New York City area during free play in a large laboratory playroom (6 × 9 m) as shown in Figure 1A. Play sessions lasted 20 min. Infants' age ranged from 10.75 to 19.53 months (*M* = 15.28) and their walking experience ranged from 0.10 to 9.01 months (*M* = 3.09). The study protocol was approved by the New York University Institutional Review Board. Infants' parents gave written consent for participation. For those parents who gave additional permission, videos from the session are shared on Databrary.org. We recorded infants' walking paths from four camera views: a fixed overhead view captured the entire playroom, two fixed cameras recorded side views of the room, and a camera held by an experimenter recorded a close-up view of the infant. The experimenter did not interact with infants or caregivers during the session.

To define the training paths for the infant-trained robots, we first identified bouts of walking. Using Datavyu (datavyu.org), a primary coder scored the onset (when infants' foot lifted off the floor) and offset of each walking bout (when infants were stationary for ≥500 ms). A second coder independently scored 25% of each session to ensure inter-observer agreement, *rs* > 0.96, *ps* < 0.001 for number of bouts, bout duration, number of steps per bout. To define the shape of each path and the angle between consecutive steps, a coder used Matlab software (DLTDataViewer; https://www.unc.edu/~thedrick/software1.html) to manually digitize the location of each step using an overhead camera view that covered the entire playroom. If infants' feet were momentarily occluded, coders estimated their location based on the preceding and following steps. We used the xy coordinates of these points to map the paths infants took through the playroom (adjusting for lens and perspective distortion). Using known distances, we verified that the digitizing method returned < 1% error per bout.

### Robot simulations

To ensure robustness, each pair of teams competed in 1,000 head-to-head matches. Because such a large number of real-world competitions is impractical, we used a computer simulation environment—RoboCup 3D—as a low cost, high efficiency alternative to real model testing (Boedecker and Asada, 2008; Xu and Vatankhah, 2013). In addition, previous work showed that walking parameters learned through RoboCup simulations can be translated to effective walking parameters for physical robots (Farchy et al., 2013). The RoboCup 3D simulation environment is based on SimSpark (http://simspark.sourceforge.net/), a generic, physical, multiagent system simulator that uses the Open Dynamics Engine library (ODE; http://www.ode.org/). The library provides rigid body dynamics with collision detection, friction, and support for the modeling of advanced motorized hinge joints used in the humanoid agents.

The robots used in the simulation are loosely modeled after the Aldebaran Nao robot (http://www.aldebaran-robotics.com). All robots have a height of 57 cm, a mass of 4.5 kg, and 22 degrees of freedom (six in each leg, four in each arm, and two in the neck). Each robot has proprioception of all joints, pressure sensors on its feet, two gyrometers, and an accelerometer. The joint perceptors and effectors enable monitoring and control of the hinge joints. Joint effectors allow the robot to specify the torque and direction in which to move.

### Robot walk engine and optimization

To walk, a request for velocity and a destination for the feet and torso are sent to a walk engine, which uses this request, together with inverse kinematic and sensor information, to determine the next desired joint positions. The engine sends these joint positions to proportional-integral-derivative (PID) controllers that convert the positions into torque commands, which are then sent to the simulator for processing.

We used an open source parameterized walk engine (MacAlpine and Stone, 2016) that first selects a path for the torso to follow, and then determines where the feet should be with respect to the torso's location. More than 40 parameters are used to calculate the position of the feet with respect to the torso. A full description of the technical and mathematical details of the walk can be found in MacAlpine et al. (2012a).

The parameters for the walk engine are initialized based on previous testing on an actual Nao robot (MacAlpine et al., 2012a). Robots that use walk engines with these values, without any further parameter optimization (i.e., training to walk), are stable but slow walkers. We refer to these robots as “no-training” and used them as a baseline. All other teams were trained through walking optimization.

In the walking optimization, we wished to improve robots' stability during various situations encountered during soccer game play and to increase their speed. In this procedure, the robot learns a set of parameters by walking toward a series of destinations on the field (goToTarget optimization sub-task; MacAlpine et al., 2012a). The robot is rewarded based on the distance traveled toward the destination. If the robot reaches a destination ahead of time, it receives extra reward based on the distance it could have traveled given the remaining time. The robot also has “stop destinations,” where it is penalized for overshooting the destination. Finally, the robot receives a penalty if it falls during the optimization run (for full equations describing the robot reward system, see MacAlpine et al., 2012a). Over the course of the optimization, robots learn to walk increasingly faster, with fewer errors. Because it is impractical to optimize all 40 parameters, we selected a subset of 25 parameters, based on their high potential impact on the speed and stability of the robots (see Tables 1, 4 for the list of selected parameters and further details in MacAlpine et al., 2012a). Moreover, because we focused on walking optimization, all phases of optimization that relate to other skills (e.g., teaching robots how to dribble or kick the ball) were similar to previous work and were held constant across teams (Urieli et al., 2011; MacAlpine et al., 2012a).

**Table 1:** Final values of optimized parameters after each training regimen in Tournament 1.

**Parameter**	**Infants**	**Square**	**Circle**	**Line**	**No-training**
Maximum size of steps (radians)	0.54	1.74	0.67	1.59	1.22
Maximum size of steps for x coordinates (mm)	78.42	157.90	135.17	201.10	50.00
Maximum size of steps for y coordinates (mm)	123.22	33.43	56.67	35.80	40.00
How much center of mass is shifted from side to side (mm)	−39.08	−23.83	−11.39	3.44	20.00
Height of the torso from ground (mm)	147.88	120.17	165.90	80.57	175.00
Maximum height of foot from ground during step (mm)	84.67	93.97	87.59	76.70	20.00
Fraction of a phase the swing foot remains still before moving	0.35	0.16	0.09	−0.08	0.20
Fraction of a phase that the swing foot on the ground before lifting	−0.12	−0.12	−0.70	−0.81	0.20
Duration of single step in seconds	0.04	0.06	0.08	0.08	0.38
Expected difference between commanded COM and sensed COM	86.22	42.35	13.47	−6.33	0.00
Factor of how fast the step sizes change per time cycle	0.06	0.06	0.07	0.07	0.03
Maximum COM error in millimeters before the steps are slowed	30.26	64.88	44.80	108.90	7.50
Maximum COM error in millimeters before all velocity reach 0	172.86	129.77	60.37	134.70	12.50
Constant offset between the torso and feet (mm)	0.99	2.33	2.26	−1.01	2.50
Factor of the step size applied to the forwards position of the torso	1.01	0.80	0.79	0.57	0.50
Angle of foot when it hits the ground in radians	0.38	1.09	0.92	1.14	0.60
Fraction of a phase that the swing foot spends in the air	1.44	1.21	1.84	1.78	0.60
Proportional controller values for the torso angles – tilt	0.13	−0.05	−0.07	−0.08	0.15
Proportional controller values for the torso angles – roll	0.05	−0.07	0.22	0.80	0.20
Proportional controller values for controlling COM (x)	1.25	1.19	0.87	0.98	1.00
Proportional controller values for controlling COM (y)	1.62	0.95	1.13	0.56	1.00
Proportional controller values for controlling COM (z)	0.10	0.22	0.03	0.39	0.00
Proportional controller values for moving arms (x)	−0.04	0.15	−0.07	−0.16	0.00
Proportional controller values for moving arms (y)	0.27	−0.15	0.14	0.56	0.00

### Soccer game procedure

We evaluated the success of each training regimen using a tournament of soccer games among teams of eleven simulated robot players. All players on a team were trained in the same way. Each team competed to get a ball into the other team's goal (Figure 1B). The games consisted of two 5-m halves (without stopping the time). Each half began with a kick-off, and all players were located on their team's side of the field.

We calculated the number of goals scored per team per match, and the number of wins in each set of 1,000 head-to-head matches. As in human soccer, the team that scored the most goals at the end of the game won. If the score was even, we declared a tie. To evaluate the success of each team (and thereby the success of its training regimen), we focused on the magnitude and consistency of their wins. The magnitude of each team's wins is expressed by their average goal difference, or the average number of goals scored relative to the number of goals conceded. Consistency is expressed by a high number of league points across the tournament. Using the standard league point system in human soccer, a team gains 3 points for a win, 1 point for a tie, and 0 points for a loss. Importantly, the motion targets used during the soccer matches are similar no matter what walk is used for training. That is, robots walk to the same target positions near the ball even if they struggle to do so given their current walking capability. Therefore, an analysis of small differences in locomotion during the matches is not informative for determining differences in functional walking. However, differences in locomotion between teams can accumulate over time to produce differences in scoring.

## Tournament 1: infant paths vs. geometric paths

### Training regimens

Our first aim was to examine the role of varied paths in learning functional walking. We compared a team trained on natural, varied infant walking paths to four teams trained on uniform, geometric walking paths. To create the infant training course, we randomly selected 15 infant play sessions. We then took the coordinates of each infant path and mapped those points onto the soccer field where each grid space is 1 × 1m^2^. For each session, we capped stationary periods at 2 s, and then randomly sampled a 4-min block of walking time plus stops. Although infants stop for longer periods, after 2 s, the robot is usually fully stabilized, so longer pauses have no additional merit. Three infants had fewer than 4 min of walking plus stops, so their paths were repeated until 4 min accumulated. Then, we concatenated the randomly sampled 4-min blocks from each of the 15 infants to create a 1-h long training course (a realistic duration for training in terms of computational time complexity). This training path was used to optimize the infant-trained team in Tournament 1. During training, the robots walked sequentially toward each step specified by infants' paths. Whenever the infant stopped walking, the robot also stopped walking and stood in place.

For the less varied training regimens, we optimized the walking engine parameters by training the robots on either a straight-line, circle, or square path. The straight-line team walked continually forward for 10 walking segments in which the robots walked for 7 s and then stopped for 2 s. The straight-line team's walking parameters were fit using the average of these 10 walking attempts. The circle team walked along a fixed-size circular path where the target heading was updated every second for 20 s and then stopped for 2 s. The square team walked once around the square before stopping for 2 s and then once around the square stopping for 2 s at each corner in alternation (the size of the square was determined by the robot's walk - 5 s of walking per side, 20 s total). Both the square team and the circle team repeated their walks 7 times. All teams' walking parameters were fit using the average of all repetitions. In previous work, the fitness values of robots trained on geometric paths plateaued after 200 generations of learning. In the current study, the duration of each training regimen was sufficient to include 300 generations of learning, thus there was no need for further training time. The final team used the initial parameters of the walk engine without any optimization (the no-training team; see Methods). After the training phase, the five teams competed in a RoboCup 3D simulation.

### Results and discussion

Overall, more variety in training led to better performance. Final values of the walking parameters (Table 1; see MacAlpine et al., 2012b for more details) indicate that training on varied paths leads to improvements in the optimization process in terms of stability (e.g., larger step size applied to the forward position of the torso, smaller foot angle at ground contact, higher proportion of stationary time for the swing foot), speed (e.g., shorter duration of single steps), and shifts in direction (e.g., smaller steps). The infant-trained team, which had the most varied paths, beat all other training regimens in terms of consistency (as measured by League points) and magnitude (as measured by average goal difference scores).

**Figure 2:**
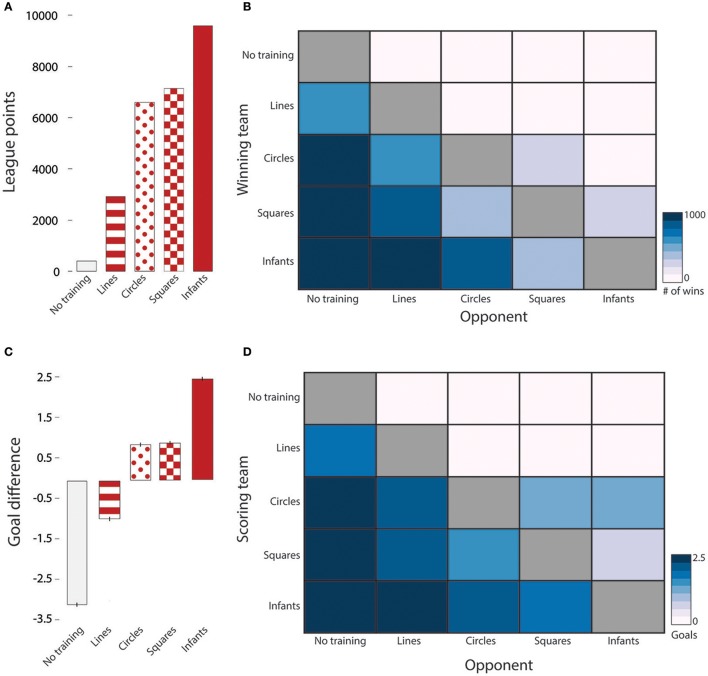
Tournament 1 results: Infant paths vs. Geometric paths. **(A)** Accumulated league points, indicating consistency of training success. **(B)** Each team's wins (rows) against all possible opponents (columns) Color denotes the number of wins and does not include ties between teams. **(C)** Average goal difference, indicating magnitude of training success. The infant-trained team scored more goals and conceded fewer than all other teams. **(D)** The average number of goals scored by each team (rows) against all other opponents (columns). The infant-trained team scored fewer goals against more variably trained teams (squares, circles).

The infant-trained team won Tournament 1 with 9,701 League points, winning 2,888 games, tying 1,037, and losing only 75. The square-trained team came in second, followed by the circle-trained team, the line-trained team, and the no-training team, respectively (Figure 2A; see Table 2 for full description of the competition results). As in previous studies (MacAlpine et al., 2012a), the no-training team never beat a trained team (0 wins, see Table 2), demonstrating the essential value of optimizing the walk engine. Figure 2B depicts the wins of each team (rows) against all possible opponents (columns). The blue gradient in the infant team row shows that as the variety of the opponent's path increased, the number of infant team wins decreased. These findings suggest that more varied training regimens generalized to the new task constraints of RoboCup and led to better functional performance.

**Table 2:** Scoring table for Tournament 1 describing results across all games.

**Team**	**League points**	**Wins**	**Losses**	**Ties**	**Goals scored (*M* ± *SE*)**	**Goals conceded (*M* ± *SE*)**
Infants	9,701	2,888	75	1,037	2.43 ± 0.04	0.02 ± 0.003
Squares	7,463	1,898	333	1,769	1.03 ± 0.02	0.09 ± 0.01
Circles	6,602	1,696	790	1,514	1.21 ± 0.03	0.25 ± 0.01
Lines	2,927	611	2,295	1,094	0.20 ± 0.01	1.14 ± 0.02
No-training	400	0	3,600	400	0 ± 0	3.36 ± 0.03

The infant-trained team also won in terms of magnitude by achieving a larger average goal difference across the tournament [Figure 2C; *F*_(4, 19995)_ = 5595.91, *p* < 0.001, one-way ANOVA on average goal difference]. As shown in Figure 2C, the infant team had the highest average goal difference followed by the circle and square teams (which did not differ, *p* = 1.00), the line team, and the no-training teams, respectively (all other Bonferroni *post-hoc* tests *ps* < 0.001). Figure 2D depicts the average number of goals scored against each possible opponent. The blue gradient in the infants' row shows that as the variety of the opponent's path increased, the number of goals infants scored decreased (see Table 3 for pairwise comparisons). Taken together, the results of Tournament 1 indicate that the variety in infants' paths is a feature that leads to better functional walking as indexed by success in robot soccer. Moreover, path variety promotes generalization to new, untrained paths.

**Table 3:** Pairwise comparisons for the average goal differences in Tournament 1.

**Competition**	***M***	***SE***	***t***	***p***
Lines v. no-training	0.79	0.02	33.74	<0.001
Circles v. no-training	3.89	0.03	117.84	<0.001
Squares v. no-training	2.91	0.03	85.31	<0.001
Infants v. no-training	5.85	0.02	306.49	<0.001
Circles v. lines	0.88	0.03	33.77	<0.001
Squares v. lines	0.94	0.03	35.54	<0.001
Infants v. lines	2.74	0.03	98.95	<0.001
Squares v. circles	0.15	0.02	9.19	<0.001
Infants v. circles	0.77	0.03	31.71	<0.001
Infants v. squares	0.25	0.02	13.21	<0.001

## Tournament 2: individual differences in the variety of infant paths

### Training regimens

Our second aim was to test whether differences in the natural variety of infant walking paths affect functional performance. To ensure that team differences in variety did not depend on the number of infants contributing to the robot-training regimen, we created 5 equal sized groups of 15 infants by clustering the paths of the 75 infants who did not contribute to the training regimen for Tournament 1. We used a k-means clustering algorithm with *k* = 5 (Spath, 1985). To maintain equal sized groups, we applied an equal cardinality constraint to the clusters while keeping them as spatially cohesive as possible (Zhu et al., 2010).

Clusters were based on variation in four interdependent aspects of walking: path shape, step direction, number of steps, and number of starts and stops. We calculated variety in *path shape* as the standard error of path curvature. For bouts of ≥4 steps, we calculated path curvature by averaging the overall path curvature (the shortest distance between the start and end points of the bout divided by the total distance traveled) and step-to-step curvature (calculated the same way from each series of 3 points in the bout). We calculated variety in *step direction* as the standard error of the change in degrees of the plane angle between each pair of steps. We calculated variety in *path length* as the standard error of the number of infant steps per walking bout. Finally, we calculated the *number of starts and stops* as the total number of bouts.

**Figure 3:**
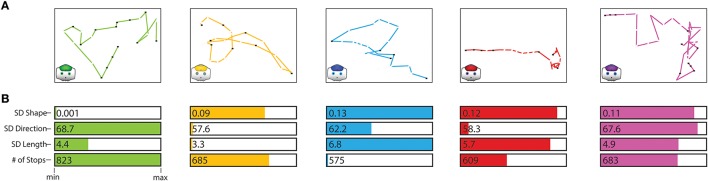
Exemplar robot training paths. **(A)** Exemplar paths from each of the five robot training courses built from clustered infants' walking paths. Colored lines show the path trajectory, dashes indicate steps, black dots indicate stops. **(B)** Bars showing relative combinations of walking features for each team's training course. Values are scaled from the minimum to the maximum across teams.

Following the same procedure used for the infant-trained team in Tournament 1, we created 5 robot-training courses using the paths of the 15 infants in each group. Thus, the robot training courses represented the combination of dimensions in each group of infant paths. Figure 3 shows the 5 infant-trained teams, distinguished by color. The green team was characterized by a high variation in step direction (*SD* of the change in degrees between each pair of steps) and a high number of stops and low variation in path shape (*SD* of path curvature) and relatively low variation in path length (*SD* of the number of steps per bout). The yellow team was characterized by relatively high variation in path shape and a high number of stops and low variation in step direction and path length. The blue team was characterized by a high variation in path shape and path length and a low number of stops and relatively low variation in step direction. The red team was characterized by high variation in path shape and length and a relatively low number of stops and low variation in step direction. The purple team had relatively high variation along all dimensions. Figure 3A depicts examples of paths from each training course. As a baseline, we trained an additional team on a straight-line training course, just as the line-trained team in Tournament 1.

### Results and discussion

Overall, natural differences in the variety of infants' paths resulted in a consistent pattern of wins and losses in RoboCup, suggesting that some combinations of variation are more beneficial for functional walking than others. Final values of the optimized walking parameters (Table 4) indicate that although all teams were trained on variable paths, variability in more aspects of walking leads to improved whole-body control (e.g., longer constant offset between the torso and the feet, higher proportion of time the swing foot spends in the air, torso higher from the ground) and faster movement (e.g., shorter duration of single steps).

**Table 4:** Final values of optimized parameters after each training regimen in Tournament 2.

**Parameter**	**Purple**	**Red**	**Blue**	**Yellow**	**Green**
Maximum size of steps (radians)	0.71	0.61	0.66	0.79	0.99
Maximum size of steps for x coordinates (mm)	91.2	73.37	71.93	76.00	93.94
Maximum size of steps for y coordinates (mm)	126.2	143.41	134.73	113.96	156.08
How much center of mass is shifted from side to side (mm)	−52.12	−27.27	−25.47	−10.13	−20.69
Height of the torso from ground (mm)	172.8	145.84	149.45	131.63	113.35
Maximum height of foot from ground during step (mm)	79.86	111.73	120.14	104.72	73.33
Fraction of a phase the swing foot remains still before moving	0.57	0.68	0.70	0.66	0.69
Fraction of a phase that the swing foot on the ground before lifting	−0.02	0.31	0.33	0.29	0.40
Duration of single step in seconds	0.04	0.06	0.06	0.06	0.05
Expected difference between commanded COM and sensed COM	−5.28	−38.29	36.42	−92.53	−20.70
Factor of how fast the step sizes change per time cycle	0.06	0.06	0.05	0.06	0.08
Maximum COM error in millimeters before the steps are slowed	6.53	15.68	26.03	25.69	−33.54
Maximum COM error in millimeters before all velocity reach 0	216.11	154.91	110.82	200.95	135.28
Constant offset between the torso and feet (mm)	4.05	1.39	−0.23	3.44	0.37
Factor of the step size applied to the forwards position of the torso	1.07	1.06	1.10	1.08	1.10
Angle of foot when it hits the ground in radians	0.77	0.59	0.64	0.67	0.48
Fraction of a phase that the swing foot spends in the air	1.32	0.88	0.90	0.94	0.68
Proportional controller values for the torso angles – tilt	0.22	−0.09	0.22	0.14	−0.40
Proportional controller values for the torso angles – roll	0.1	0.01	0.20	0.01	−0.08
Proportional controller values for controlling COM (x)	1.8	1.19	1.37	1.28	1.59
Proportional controller values for controlling COM (y)	0.18	0.63	0.64	1.16	0.65
Proportional controller values for controlling COM (z)	0.01	0.1	0.09	0.17	0.25
Proportional controller values for moving arms (x)	0.38	−0.31	−0.38	−0.05	−0.04
Proportional controller values for moving arms (y)	0.57	0.16	0.57	0.44	0.27

**Figure 4:**
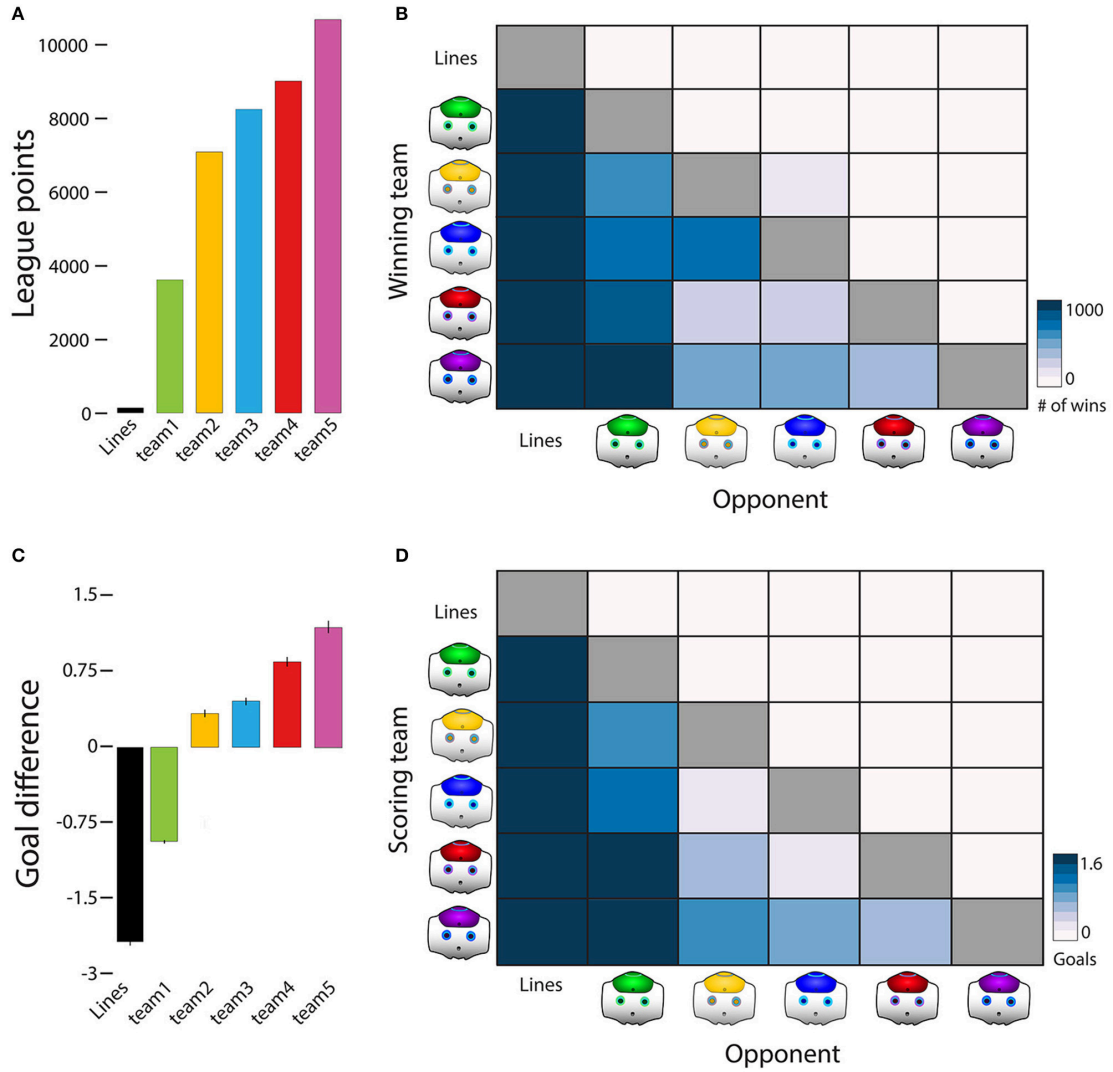
Tournament 2 results: individual differences in infant paths. **(A)** Accumulated league points, indicating consistency of training success. **(B)** Each team's wins (rows) against all possible opponents (columns). Color denotes the number of wins and does not include ties between teams. **(C)** Average goal difference, indicating magnitude of training success. The purple team scored more goals and conceded fewer than all other teams. **(D)** The average number of goals scored by each team (rows) against all other opponents (columns). Teams that had high variability in path shape, step direction, and bout length, and had a higher number of starts and stops were more likely to win.

The purple-trained team won Tournament 2, with 11,786 League points, winning 3,420 games, tying 1,526, and losing only 54. The red-trained team came in second, followed by the blue-trained team, yellow-trained team, green-trained team, and the line-trained team. As expected, the line-trained team performed worse than any team trained on infant paths. The line-trained team never beat an infant-trained team, scored no goals, and accumulated 155 ties (see Table 5 and number of league points in Figure 4A). Figure 4B depicts the wins of each team (rows) against all possible opponents (columns). The blue gradients across rows show the patterns of wins and losses. The win/loss matrix is not symmetrical because teams may tie.

**Table 5:** Scoring table for Tournament 2 describing results across all games.

**Team**	**League points**	**Wins**	**Losses**	**Ties**	**Goals scored (*M* ± *SE*)**	**Goals conceded (*M* ± *SE*)**
Purple	11,786	3,420	54	1,526	1.46 ± 0.02	0.02 ± 0.001
Red	9,307	2,407	507	2,086	0.89 ± 0.02	0.13 ± 0.01
Blue	8,052	1,982	912	2,106	0.74 ± 0.01	0.23 ± 0.01
Yellow	7,561	1,795	1,029	2,176	0.63 ± 0.01	0.26 ± 0.01
Green	3,655	912	3,169	919	0.30 ± 0.01	1.15 ± 0.02
Lines	155	0	4,845	155	0 ± 0	2.26 ± 0.01

The purple-trained team also had the highest average goal difference across the tournament [*F*_(5, 29994)_ = 5281.72, *p* < 0.001, one-way ANOVA on average goal difference]. As shown in Figure 4C, the purple-trained team was followed by the red-trained team, the blue-trained team, the yellow trained team, the green-trained team, and the line-trained team, respectively (all Bonferroni *post-hoc* tests *p*s < 0.001). Figure 4D shows the goals scored (rows) and conceded (columns) for each set of competitions (see Table 6 for pairwise comparisons). Taken together, the results from Tournament 2 suggest that teams trained on a training course with high variability across most features fared better than teams trained on a course that had low variability on at least one feature.

**Table 6:** Pairwise comparisons for the average goal differences in Tournament 2.

**Competition**	***M***	***SE***	***t***	***p***
Green v. lines	1.51	0.03	59.11	<0.001
Yellow v. lines	2.06	0.03	71.55	<0.001
Blue v. lines	2.25	0.03	83.57	<0.001
Red v. lines	2.39	0.03	89.93	<0.001
Purple v. lines	3.10	0.03	105.55	<0.001
Yellow v. green	0.84	0.03	32.13	<0.001
Blue v. green	1.13	0.03	41.09	<0.001
Red v. green	1.39	0.03	48.40	<0.001
Purple v. green	2.36	0.03	68.52	<0.001
Blue v. yellow	0.06	0.02	2.93	0.003
Red v. yellow	0.26	0.02	12.66	<0.001
Purple v. yellow	0.69	0.02	28.79	<0.001
Red v. blue	0.20	0.02	10.68	<0.001
Purple v. blue	0.20	0.02	28.68	<0.001
Purple v. red	0.41	0.02	18.60	<0.001

## General discussion

We combined the power of robotic modeling with the power of behavioral observation in infancy research. Specifically, we tested the functional utility of varied paths in infant walking using simulated soccer-playing robots, a model that shares many of the critical components of real-world infant walking (embodied agents moving purposefully through a changing environment). We found that optimizing simulated robot walking using more varied paths in a solitary, uniform training environment led to better functional outcomes in the new context of soccer, where the robots moved through a changeable environment filled with other agents. We suggest that infants' early experience with varied walking paths constitutes a natural training set that is a feature—not a bug—of learning functional walking.

### The importance of variety for functional performance: tournament 1

With a changing body in a changing environment, learning fixed motor solutions is maladaptive (Adolph and Robinson, 2015). Instead, infants must learn to tailor their motor actions to dynamic body-environment relations. Indeed, experienced walking infants display tremendous flexibility and generativity. They distinguish safe from risky ground within two degrees of slant while navigating slopes, and one centimeter of accuracy while crossing drop-offs, gaps, and bridges (for reviews, see Adolph and Robinson, 2015; Adolph and Franchak, 2016). They update their assessment of whether slopes are walkable to take heavy shoulder-packs or slippery-soled shoes into account (Adolph and Avolio, 2000; Adolph et al., 2010). They modify their walking patterns (e.g., by altering step length and velocity) while approaching and crossing obstacles (Gill et al., 2009; Kretch and Adolph, 2017). And they find new solutions on the fly such as scooting down steep slopes, backing down drop-offs, and using handrails to cross narrow bridges (Adolph and Robinson, 2015).

How do infants learn such flexible, functional motor behaviors? A central principle in motor control is that variable practice minimizes the tendency to learn a fixed motor solution for a specific motor problem and encourages generalization to new variants of the task (Schmidt, 1975). But few laboratory training studies have focused on infant motor skill acquisition, and none involved a training regimen comparable to the magnitude and variety of infants' everyday walking experiences. Outside the laboratory, the flux of everyday life is replete with varied walking paths, varied footwear and clothing, varied ground surfaces and layouts, and varied tasks and activities. Infants' natural walking experience—“variable practice” writ large—may ensure that they learn flexible rather than fixed behaviors.

In the current studies, varied practice was operationally defined as variations in walking paths. Accordingly, in Tournament 1, teams with no training, or teams trained to walk along a straight line performed worst. Their narrow experiences did not prepare them to deal with the variety of movements needed to succeed in soccer. Robots trained on more varied paths (circles, squares) faired better. These teams had more experience turning, controlling the two sides of the body differently, and stopping to change direction. The infant-trained team experienced the most varied paths and performed best. Experiencing a wider variety of paths during training better led to more functional and adaptive performance in soccer.

### Variety is a feature of learning to walk in infants: tournament 2

Every infant walking path was varied, and each dimension of variation was present in every robot team. However, because the dimensions are interdependent, high variability on all dimensions is unlikely. For example, a high number of stops likely limits the number of steps in a path, and consequently limits the variability in path shape and step direction. This interdependence among aspects of path variation is a fundamental characteristic of infant walking. Thus, no single feature of variation can explain the pattern of results in Tournament 2, and no single feature was more important than any other. Instead, the relative combination of dimensions differed among training regimens and these differences were crucial for functional performance. Teams that performed best showed high variability on multiple dimensions of path variation and did not show low variability on any dimension. It is important to note that we tested *variability* in path features and not the average values. For example, high variability in path curvature does not imply more curved bouts overall, but rather a wide range of path shapes—some that were straighter and some that were more curved. Findings from Tournament 2 show that varied experience with multiple walking dimensions results in better functional walking.

Is training on the most varied infant paths sufficient to beat the current RoboCup world champions? Possibly. The winning 2017 robot soccer team, “UT-Austin Villa,” in the relevant division (3D simulation league) was also optimized for varied walking using a hand selected training course (MacAlpine and Stone, 2018). There are many ways to manipulate training paths to optimize variability. Future work should investigate which specific aspects of variable walking helped our infant team outscore the geometrically trained teams. Simulated “infant-based” training paths that isolate one aspect of variability may help to parse the necessarily interdependent aspects of variability found in real infant walking paths. Future studies along these lines may provide important insights for AI researchers and roboticists about how to improve walking in robots. Regardless, our findings focus on infants and suggest that their everyday walking experience serves as useful training set for functional walking. Through incidental learning in the course of free play, infants likely learn to walk using a highly adaptive natural training regimen.

## Conclusion

What is the best way to learn a generative skill, like functional walking? Answers to this kind of developmental question require appropriate models. Walking and other flexible, adaptive motor skills develop in real bodies, performing real tasks, in real environments. Robots are good models for development because they, like infants, must learn to cope with a body embedded in an environment (Adolph and Robinson, 2015). Similarly, RoboCup is a good domain to test functional walking performance because it requires robots to update their actions in response to a dynamically changing environment. Using robots allowed us to demonstrate that variety in everyday spontaneous activity leads to improved functional performance. Reciprocally, we suggest that AI researchers may benefit by observing everyday learning in human infants and other animals that acquire functional, adaptive performance.

## Author note

A portion of this work took place at New York University, and was supported by NICHD grant # R37HD033486 to KA. A portion of this work took place in the Learning Agents Research Group (LARG) at UT Austin, and was supported by NSF (CNS-1305287, IIS-1637736, IIS-1651089, IIS-1724157), Intel, Raytheon, and Lockheed Martin awards to PS. PS serves on the Board of Directors of Cogitai, Inc. Human subjects participation was approved by the NYU IRB-FY2016-825. The terms of this arrangement have been reviewed and approved by the University of Texas at Austin in accordance with its policy on objectivity in research. We are grateful to Do Kyeong Lee, Orit Herzberg-Keller, Carli Heiman, Joshua Schneider, Rose Egan, and Sinclaire O'Grady for their help with data coding and processing.

## Author contribution

All authors listed, have made substantial, direct and intellectual contribution to the work, and approved it for publication.

### Conflict of interest statement

The authors declare that the research was conducted in the absence of any commercial or financial relationships that could be construed as a potential conflict of interest.

## References

[B1] Adolph K. E. (2008). Learning to move. Curr. Dir. Psychol. Sci. 17, 213–218. 10.1111/j.1467-8721.2008.00577.x 19305638 PMC2658759

[B2] Adolph K. E. Avolio A. M. (2000). Walking infants adapt locomotion to changing body dimensions. J. Exp. Psychol. Hum. Percept. Perf. 26, 1148–1166. 10.1037/0096-1523.26.3.1148 10884014

[B3] Adolph K. E. Cole W. G. Komati M. Garciaguirre J. S. Badaly D. Lingeman J. M.. (2012). How do you learn to walk? Thousands of steps and dozens of falls per day. Psychol. Sci. 23, 1387–1394. 10.1177/0956797612446346 23085640 PMC3591461

[B4] Adolph K. E. Franchak J. M. (2016). The development of motor behavior. WIREs Cogn. Sci. 8:e1430. 10.1002/wcs.1430 27906517 PMC5182199

[B5] Adolph K. E. Karasik L. B. Tamis-LeMonda C. S. (2010). Using social information to guide action: infants' locomotion over slippery slopes. Neural Netw. 23, 1033–1042. 10.1016/j.neunet.2010.08.012 20875725 PMC2963195

[B6] Adolph K. E. Robinson S. R. (2015). Motor development, in Handbook of Child Psychology and Developmental Science, 7th Edn, Vol. 2 Cognitive Processes, eds Liben L. Muller U. (New York, NY: Wiley), 114–157.

[B7] Adolph K. E. Vereijken B. Shrout P. E. (2003). What changes in infant walking and why. Child Dev. 74, 475–497. 10.1111/1467-8624.7402011 12705568

[B8] Bernstein N. A. (1996). On dexterity and its development, in Dexterity and its Development, eds Latash M. L. Turvey M. T. (Mahwah, NJ: Erlbaum), 3–244.

[B9] Bisi M. C. Stagni R. (2015). Evaluation of toddler different strategies during the first six-months of independent walking: a longitudinal study. Gait Posture 41, 574–579. 10.1016/j.gaitpost.2014.11.017 25636708

[B10] Boedecker J. Asada M. (2008). Simspark - concepts and application in the 3d soccer simulation league, in Workshop Proceedings of International Conference on Simulation, Modeling, and Programming for Autonomous Robots (Venice), 174–181.

[B11] Bonneuil N. Bril B. (2012). The dynamics of walking acquisition: a tutorial. Infant Behav. Dev. 35, 380–392. 10.1016/j.infbeh.2012.05.001 22721738

[B12] Bril B. Dupuy L. Dietrich G. Corbetta D. (2015). Learning to tune the antero-posterior propulsive forces during walking: a necessary skill for mastering upright locomotion in toddlers. Exp. Brain Res. 233, 2903–2912. 10.1007/s00221-015-4378-6 26246420

[B13] Burkhard H.-D. Duhaut D. Fujita M. Lima P. Murphy R. Rojas R. (2002). The road to RoboCup 2050. IEEE Robot. Automat. Magazine 9, 31–38. 10.1109/MRA.2002.1019488

[B14] Cangelosi A. Schlesinger M. Smith L. B. (2015). Developmental Robotics: From Babies to Robots. London: MIT Press.

[B15] Catalano J. F. Kleiner B. M. (1984). Distant transfer in coincident timing as a function of variability of practice. Percept. Mot. Skills 58, 851–856. 10.2466/pms.1984.58.3.851

[B16] Chang C. L. Kubo M. Buzzi U. Ulrich B. (2006). Early changes in muscle activation patterns of toddlers during walking. Infant Behav. Dev. 29, 175–188. 10.1016/j.infbeh.2005.10.001 17138273 PMC1550343

[B17] Cherng R. J. Liu C. F. Lau T. W. Hong R. B. (2007). Effect of treadmill training with body weight support on gait and gross motor function in children with spastic cerebral palsy. Am. J. Phys. Med. Rehabilitat. 86, 548–555. 10.1097/PHM.0b013e31806dc302 17581289

[B18] Clark J. E. Whitall J. Phillips S. J. (1988). Human interlimb coordination: The first 6 months of independent walking. Dev. Psychobiol. 21, 445–456. 10.1002/dev.420210504 3402667

[B19] Davids K. Bennett S. Newell K. M. (2006). Movement System Variability. Champaign, IL: Human Kinetics.

[B20] Farchy A. Barrett S. MacAlpine P. Stone P. (2013). Humanoid robots learning to walk faster: from the real world to simulation and back, in Proceedings of the 2013 International Conference on Autonomous Agents and Multi-Agent Systems (Saint Paul, MN: International Foundation for Autonomous Agents and Multiagent Systems), 39–46.

[B21] Gibson J. J. (1979). The Ecological Approach to Visual Perception. Boston, MA: Houghton Mifflin Company.

[B22] Gill S. V. Adolph K. E. Vereijken B. (2009). Change in action: how infants learn to walk down slopes. Dev. Sci. 12, 888–902. 10.1111/j.1467-7687.2009.00828.x 19840044 PMC2769020

[B23] Gómez G. Lungarella M. Eggenberger Hotz P. Matsushita K. Pfeifer R. (2004). Simulating development in a real robot: on the concurrent increase of sensory, motor, and neural complexity, in Proceedings of the 4th Int. Workshop on Epigenetic Robotics (Genoa), 1–24.

[B24] Hallemans A. De Clercq D. Aerts P. (2006). Changes in 3D joint dynamics during the first 5 months after the onset of independent walking: a longitudinal follow-up study. Gait Posture 24, 270–279. 10.1016/j.gaitpost.2005.10.003 16314099

[B25] Hoch J. Rachwani J. Adolph K. E. (2017). Why do infants move? Locomotor exploration is more random than destination directed, in Paper presented at the meeting of the Society for Research in Child Development (Austin, TX).

[B26] Ivanenko Y. P. Dominici N. Cappellini G. Dan B. Cheron G. Lacquaniti F. (2004). Development of pendulum mechanism and kinematic coordination from the first unsupported steps in toddlers. J. Exp. Biol. 207, 3797–3810. 10.1242/jeb.01214 15371487

[B27] Ivanenko Y. P. Dominici N. Lacquaniti F. (2007). Development of independent walking in toddlers. Exerc. Sport Sci. Rev. 35, 67–73. 10.1249/JES.0b013e31803eafa8 17417053

[B28] Kitano H. Asada M. Kuniyoshi Y. Noda I. Osawa E. (1997). RoboCup: the robot world cup initiative. Autonomous Agents 97, 340–347. 10.1145/267658.267738

[B29] Kretch K. S. Adolph K. E. (2017). The organization of exploratory behaviors in infant locomotor planning. Dev. Sci. 20, 1–17. 10.1111/desc.12421 27147103 PMC5097037

[B30] Lee D. K. Cole W. G. Golenia L. Adolph K. E. (2017). The cost of simplifying complex developmental phenomena: a new perspective on learning to walk. Dev. Sci. [Epub ahead of print]. 10.1111/desc.12615 29057555 PMC5911424

[B31] MacAlpine P. Barrett S. Urieli D. Vu V. Stone P. (2012a). Design and Optimization of an Omnidirectional Humanoid Walk: A Winning Approach at the RoboCup 2011 3D Simulation Competition, Vol. 1. Palo Alto, CA: Association for the Advancement of Artificial Intelligence.

[B32] MacAlpine P. Stone P. (2016). UT Austin Villa RoboCup 3D Simulation Base Code Release. RoboCup 2016: Robot Soccer World Cup XX.

[B33] MacAlpine P. Stone P. (2018). Overlapping layered learning. Artif. Intell. 254, 21–43. 10.1016/j.artint.2017.09.001

[B34] MacAlpine P. Urieli D. Barrett S. Kalyanakrishnan S. Barrera F. Lopez-Mobilia A. (2012b). UT Austin Villa 2011: a champion agent in the RoboCup 3D soccer simulation competition, in Proceedings of the 11th International Conference on Autonomous Agents and Multiagent Systems, Vol. 1 (Valencia: International Foundation for Autonomous Agents and Multiagent Systems), 129–136.

[B35] Moxley S. E. (1979). Schema: the variability of practice hypothesis. J. Mot. Behav. 11, 65–70. 10.1080/00222895.1979.10735173 15186973

[B36] Newell K. M. (1986). Constraints on the development of coordination, in Motor Development in Children: Aspects of Coordination and Control, eds Wade M. G. Whiting H. T. A. (Dordrecht: Nijhoff), 341–360.

[B37] Ranganathan R. Newell K. M. (2013). Changing up the routine: intervention-induced variability in motor learning. Exerc. Sport Sci. Rev. 41, 64–70. 10.1097/JES.0b013e318259beb5 23072823

[B38] Reisman D. S. Wityk R. Silver K. Bastian A. J. (2009). Split-belt treadmill adaptation transfers to overground walking in persons poststroke. Neurorehabil. Neural Repair 23, 735–744. 10.1177/1545968309332880 19307434 PMC2811047

[B39] Schmidt R. A. (1975). A schema theory of discrete motor skill learning. Psychol. Rev. 82, 225–260. 10.1037/h0076770

[B40] Schmidt R. A. (2003). Motor schema theory after 27 years: reflections and implications for a new theory. Res. Q. Exerc. Sport 74, 366–375. 10.1080/02701367.2003.10609106 14768837

[B41] Spath H. (1985). The Cluster Dissection and Analysis Theory FORTRAN Programs Examples. Chichester: Prentice-Hall, Inc.

[B42] Ulrich D. A. Lloyd M. C. Tiernan C. W. Looper J. E. Angulo-Barroso R. M. (2008). Effects of intensity of treadmill training on developmental outcomes and stepping in infants with Down syndrome: a randomized trial. Phys. Ther. 88, 114–122. 10.2522/ptj.20070139 17940103

[B43] Urieli D. MacAlpine P. Kalyanakrishnan S. Bentor Y. Stone P. (2011). On optimizing interdependent skills: A case study in simulated 3D humanoid robot soccer, in Paper presented at the Proceedings of the 10th International Conference on Autonomous Agents and Multiagent Systems (Richland, SC).

[B44] Van Rossum J. H. (1990). Schmidt's schema theory: the empirical base of the variability of practice hypothesis: a critical analysis. Hum. Mov. Sci. 9, 387–435. 10.1016/0167-9457(90)90010-B

[B45] Visser U. Burkhard H.-D. (2007). RoboCup: 10 years of achievements and future challenges. AI Magazine 28, 115–132. 10.1609/aimag.v28i2.2044

[B46] Willoughby K. L. Dodd K. J. Shields N. Foley S. (2010). Efficacy of partial body weight–supported treadmill training compared with overground walking practice for children with cerebral palsy: a randomized controlled trial. Arch. Phys. Med. Rehabil. 91, 333–339. 10.1016/j.apmr.2009.10.029 20298820

[B47] Xu Y. Vatankhah H. (2013). Simspark: An Open Source Robot Simulator Developed by the Robocup Community. Berlin; Heidelberg: Springer.

[B48] Zhu S. Wang D. Li T. (2010). Data clustering with size constraints. Knowledge Based Systems 23, 883–889. 10.1016/j.knosys.2010.06.003

